# A Systematic Review of Health Economic Analyses of Housing Improvement Interventions and Insecticide-Treated Bednets in the Home

**DOI:** 10.1371/journal.pone.0151812

**Published:** 2016-06-01

**Authors:** Frank Pega, Nick Wilson

**Affiliations:** Burden of Disease Epidemiology, Equity and Cost-Effectiveness Programme, University of Otago, Wellington, PO Box 7343, Wellington, New Zealand; University College London, UNITED KINGDOM

## Abstract

**Background:**

Housing improvements have considerable potential for improving health. So does the provision of insecticide-treated bednets for malaria prevention. Therefore we aimed to conduct updated systematic reviews of health economic analyses in both these intervention domains.

**Methods and findings:**

The search strategy included economic analyses of housing improvement interventions and use of insecticide-treated bednets for community-dwelling, healthy populations (published between 1 January 2000 and 15 April 2014). We searched the Cochrane Database of Systematic Reviews, MEDLINE, PubMed, EMBASE, and three health economics databases. Thirty-five economic analyses of seven types of intervention fulfilled the inclusion criteria. Most included studies adopted a health sector perspective and were cost-effectiveness analyses using decision analytic modeling or conducted alongside trials. The overall quality of the studies was generally likely to be adequate for informing policy-making (albeit with limitations in some areas). There was fairly consistent evidence for the cost-effectiveness/favorable cost-benefit of removing indoor lead to prevent lead poisoning and sequelae, and retrofitting insulation to prevent lung disease. But the value of assessing and improving home safety and providing smoke alarms to prevent injuries was more mixed and the economic evidence was inconclusive or insufficient for: home ventilation to prevent lung disease, installing heaters to prevent lung disease and regulating tap water temperatures to prevent scalding. Few studies (n = 4) considered health equity. The 12 studies of providing insecticide-treated bednets or hammocks to prevent malaria found these interventions to be moderately to highly cost-effective.

**Conclusions:**

This systematic review provides updated evidence that several housing improvement interventions (such as removing indoor lead and retrofitting insulation) and also the provision of insecticide-treated bednets are cost-effective interventions. Nevertheless, for some interventions additional analyses are required to better clarify their health economic and health equity value.

## Introduction

Housing quality is an important social determinant of health [1]. Thus, interventions that improve housing quality have the potential to improve individual and population health [1]. If these interventions are targeted at populations disadvantaged by living in housing of relatively poor quality, then they also have the potential for improving health equity [1]. Recent systematic review evidence suggested that selected housing interventions effectively improve health [2]. It was concluded, for example, that “housing investment which improves thermal comfort in the home can lead to health improvements, especially where the improvements are targeted at those with inadequate warmth” (p. 2) [2]. Thus, the effectiveness of some housing interventions is now fairly evidence-based [2]. In addition, some such interventions also have considerable non-health benefits in domains such as climate change, energy use and income. For example, retrofitting insulation to improve thermal comfort may not only improve health, but at the same time also reduce domestic energy use and anthropogenic green-house gas emissions.

Economic analysis is “the comparative analysis of alternative courses of action in terms of both their costs and their consequences” (p. 1) [3]. Health economic analyses encompass three primary types of studies, i.e., cost-benefit, cost-effectiveness and cost-utility analyses. The World Health Organization (WHO) has noted the central importance of evidence from economic analyses of the health impact of interventions addressing the social determinants of health, including housing-related interventions, in making the economic case for such interventions. The WHO has also noted the scarcity of and called for additional such evidence to be produced [4].

Systematic reviews of health economic analyses remain scarce, but are increasingly gaining attention. While specific, standard methodology for such systematic reviews has been proposed, additional development and refinement to these methods is required [5]. Best practice guidelines[5] are clear that such systematic reviews should always review both economic analyses conducted alongside randomized controlled trials [6] (or, more broadly, intervention studies) and economic analyses using decision analytic modeling [7]. The main use of such systematic reviews is to inform decision model development; identify the most relevant study for a specific decision context; and understand the key economic trade-offs and causal relationships in a decision model or treatment area [5]. Because cost-effectiveness analytic evidence is commonly highly context specific, systematic reviews of economic evaluations of the health impact of interventions may have relatively little potential to produce (pooled) cost-effectiveness estimates that are generalizable[5], unless multiple methodologically and statistically homogenous studies from a comparable context are available for meta-analysis.

We identified only one previous systematic review specifically of economic analyses of the health impact of housing improvements [8]. This review synthesized evidence from economic analyses that were included in another systematic review [9] of the effectiveness of housing interventions that was published in 2009 (which covered studies published before 2008). It identified two such studies of community-dwelling, healthy populations conducted before 2008, namely one study each of installing heaters [10] and retrofitting insulation [11–13], plus one study [14, 15] of a population with pre-existing conditions and another study [16] of an intervention that combined housing with major non-housing components. The review authors concluded that there is a “near absence of economic evaluation of housing improvements” (p. 843) [8]. Similarly, the authors of a cost-effectiveness study of enhanced home ventilation published in 2011 have argued that their study was only the second comprehensive economic analysis of a housing intervention [17].

An updated systematic review is therefore necessary for establishing the status of evidence from economic analyses of the health impact of housing interventions for four reasons. First, research activity on housing and health has grown substantially in the last seven years (i.e., 2008–2014), and so new studies may have been published that the previous systematic review [8] did not include. Second, this previous systematic review [8] was fairly narrow in scope for included study types and interventions. For example, it excluded any economic analyses using decision analytic modeling [7] and may also have excluded some types of housing interventions altogether, such as home safety assessment and modification (HSAM) interventions (e.g., those for preventing injuries in community-dwelling older adults). Third, this previous systematic review [8] did not search dedicated health economics databases. Furthermore, the status of equity analysis conducted as part of economic analyses has not specifically been reviewed before, despite the WHO calling specifically for economic analyses of social determinants of health interventions to assess and value effects on health equity [4].

Another in-house intervention is the provision of insecticide-treated bednets (ITBNs) for malaria prevention. This intervention has existing evidence for cost-effectiveness from a systematic review [18] that covered interventions published between 2000 and 2010. But since this is an active area for on-going research, we considered it appropriate to consider an updated review of this literature.

Given the above, our study objective was to provide updated systematic reviews of health economic analyses of both housing interventions and the provision of insecticide-treated bednets for community-dwelling, healthy populations.

## Methods

### Study eligibility criteria

We developed a strict study protocol before we commenced the search stage, which is available from the authors on request. To be included in this systematic review a study had to be an economic analysis, i.e., a cost-benefit, cost-effectiveness and/or cost-utility analysis. Following best practice guidelines [5], we included both economic analyses conducted alongside randomized controlled trials [6] (or other intervention studies) and economic analyses using decision analytic modeling [7]. Any other study types, including studies of costs alone, were excluded.

Interventions were included if they were structural, physical changes to housing infrastructure and/or contents, or involved insecticide-treated bednets (ITBNs) or insecticide-treated hammocks (ITHs). Other included in-home health promoting interventions were smoke-alarms, and devices which removed health-hazards such as unflued gas heaters or open-fire cooking stoves. Interventions that removed lead from the home (e.g., lead paint) were also included. However, interventions removing radon from homes were excluded. This was because a detailed review of the cost-effectiveness of indoor radon control interventions has previously been published by the World Health Organization [19]. We excluded interventions for changing the outdoor physical environment (e.g., creation of parks and gardens); the social environment (e.g., neighborhood crime reduction) or economic environment of the house (e.g., improve affordability of housing). We also excluded multi-mode interventions that combined structural housing interventions with more predominant other interventions (e.g., HSAM provided as a minor intervention alongside major education or exercise programs). Rehousing interventions (e.g., for low-income families) were also excluded, because they may have acted primarily through the changed social environment, rather than a change to the physical environment.

All types of static, physical, permanent houses were included. The exception was that we excluded institutionalized housing such as hospitals or supported housing for vulnerable populations. Non-static, (potentially) non-permanent houses such as caravans and house boats were also excluded, because they may not be comparable to the houses included in the systematic review.

Included participants were community-dwelling (non-institutionalized), healthy (without major pre-existing conditions) populations residing in any country. Homeless people and people with major pre-existing conditions such as moderate or severe asthma, severe visual impairments and HIV and/or AIDS were excluded from the review, because housing interventions may have a different cost-effectiveness in these populations than in the healthy general population. However, general population samples that included some people with pre-existing conditions were included. Records written in any language were included.

### Search and screening

**Fig 1:**
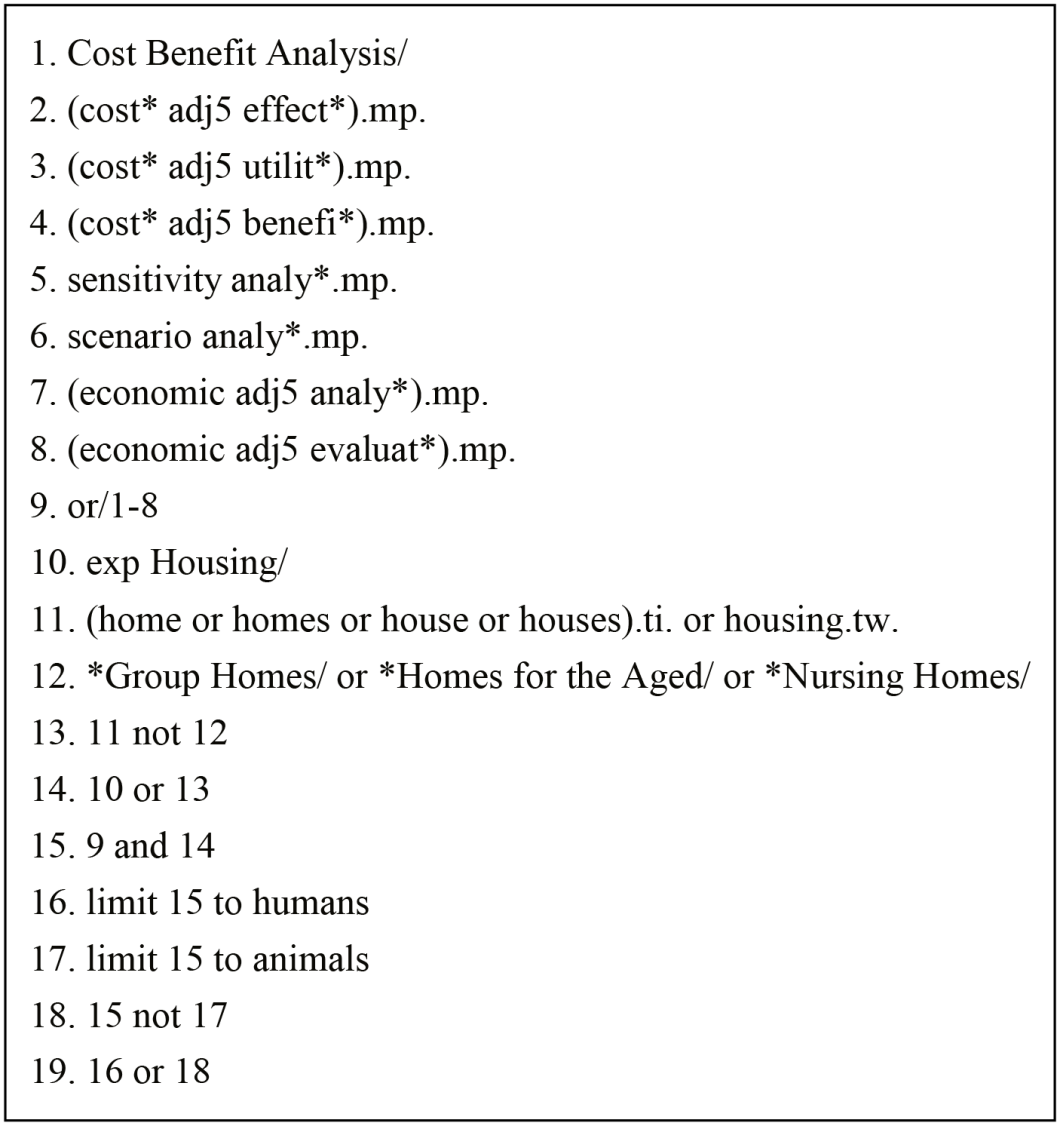
Medline search strategy.

One review author (FP) searched a total of seven electronic academic databases between 28 April and 15 May 2014 for economic analyses of the health impact of housing interventions in community-dwelling, healthy populations published between 1 January 2000 and 15 April 2014. Fig 1 presents the MEDLINE search strategy, and this search strategy was adapted to suit the searches of the other database. The Cochrane Database of Systematic Reviews (*The Cochrane Library* 2014, Issue 5), MEDLINE, PubMed, and EMBASE electronic academic databases were searched. Three specialized databases of health economic analyses were also searched, i.e., the Health Economic Evaluation Database, the NHS Economic Evaluation Database and the Tufts Cost-Effectiveness Analysis Registry. We also screened the first 30 hits on Internet search engine Google Scholar. When we were near the completion of the systematic review we searched the PubMed database again for records published after 15 May 2014 to identify any additional studies and records published since the original searches had been conducted.

One review author (FP) screened the titles of all potentially relevant records and then the abstracts of all records with potentially relevant titles. Both review authors then independently screened the full texts of all potentially relevant records in depth for the inclusion criteria. We also independently hand searched the reference lists of relevant previous systematic reviews [8, 18] and of each included study record for additional studies or records.

### Study characteristics

We followed the PRISMA guidelines [20] in reporting this systematic review. One review author (FP) conducted and the second review author (NW) double-checked the data extraction and documentation of the study and methodological characteristics of the included studies. We documented as study characteristics the setting, base year of cost data and study population. We documented as methodological characteristics the type of economic analysis (cost-benefit, cost-effectiveness and/or cost-utility analysis); study design (experimental, observational, mode-based or alongside intervention implementation); study perspective (health system or societal); time horizon; and effectiveness outcome measure (quality-adjusted life-years [QALY] gained, disability-adjusted life-years [DALY] averted, life-years saved [LYS], lives saved or deaths averted and other outcomes). We also documented as qualitative characteristics the sources for estimation of effectiveness, sources for estimation of resource utilization, discount rates used and whether sensitivity analysis had been conducted. Sources for estimation of effectiveness and resource utilization were classified as primary data collection (for example, questionnaires or trials), secondary data collection (for example, administrative records), literature (for example, systematic reviews of effectiveness) or expert opinion. Use of discount rates was classified into use on costs, effects or both costs and effects, or not used at all.

### Quality assessment

Both review authors independently assessed the quality of the included economic analyses using Drummond and Jefferson’s established checklist [21] as modified by Zelle and Baltussen [22]. This checklist included 29 items covering five categories, namely study design; effectiveness estimation; cost estimation; analysis; and interpretation of results. We independently assessed, for each study, each item along a three-point scale, allowing grading as to whether the respective item was fully considered (assigning 2 points), partially considered (1 point) or not considered (0 points) in the study. Items that were irrelevant for a study were excluded from the quality assessment. For example, if a study sourced effectiveness estimates from an individual study, then we did not assess whether the study provided details of the method of synthesis or meta-analysis of effectiveness estimates (item 11). The two reviewers discussed any disagreement between the quality assessments until resolution was reached. To provide a mean quality score, scores were summed and compared to the maximum attainable score, both for each assessment category and overall. We also screened all studies for potential disclosed and undisclosed financial conflicts of interest and note these as part of our quality assessment. In the absence of best practice guidelines for assessing publication bias in systematic review of economic analyses, we assessed whether the body of evidence included a plausible range of cost-effectiveness estimates across the included studies.

### Study findings

We extracted and documented each study’s objective, the comparator intervention(s) and the main outcomes measures (i.e., cost-effectiveness or cost-benefit measures). The main outcome measures of this systematic review were (i) the study author’s or authors’ conclusion of the cost-effectiveness or cost-benefit of the housing intervention (e.g., differentiating not, likely or highly cost-effective, or cost-saving) and (ii) the average cost per relevant health outcome (e.g., average cost per DALY averted). If study authors did not reach a clear conclusion on the cost-effectiveness of an examined housing intervention, we judged cost-effectiveness ourselves. We based our judgment on the WHO standard [23] of one per-capita gross domestic product (GDP; sourced from the World Bank [24]) per QALY gained or DALY averted as indicating high cost-effectiveness and two to three per capita GDP as indicating likely cost-effectiveness. In the absence of a global standard of cost-effectiveness for the cost of one malaria infection averted, we applied an arbitrary cost-effectiveness threshold of US$50. If a study reported cost-effectiveness or cost-benefit measures for multiple health outcomes, then we prioritized measures for the health outcomes in the following order: DALYs averted over QALYs averted over LYS over lives saved or deaths averted over other outcomes. For studies that report results from two types of economic analyses (i.e., combined cost-effectiveness and cost-benefit analyses), we extracted and reported findings from both economic analyses types.

## Results

### Search results

**Fig 2:**
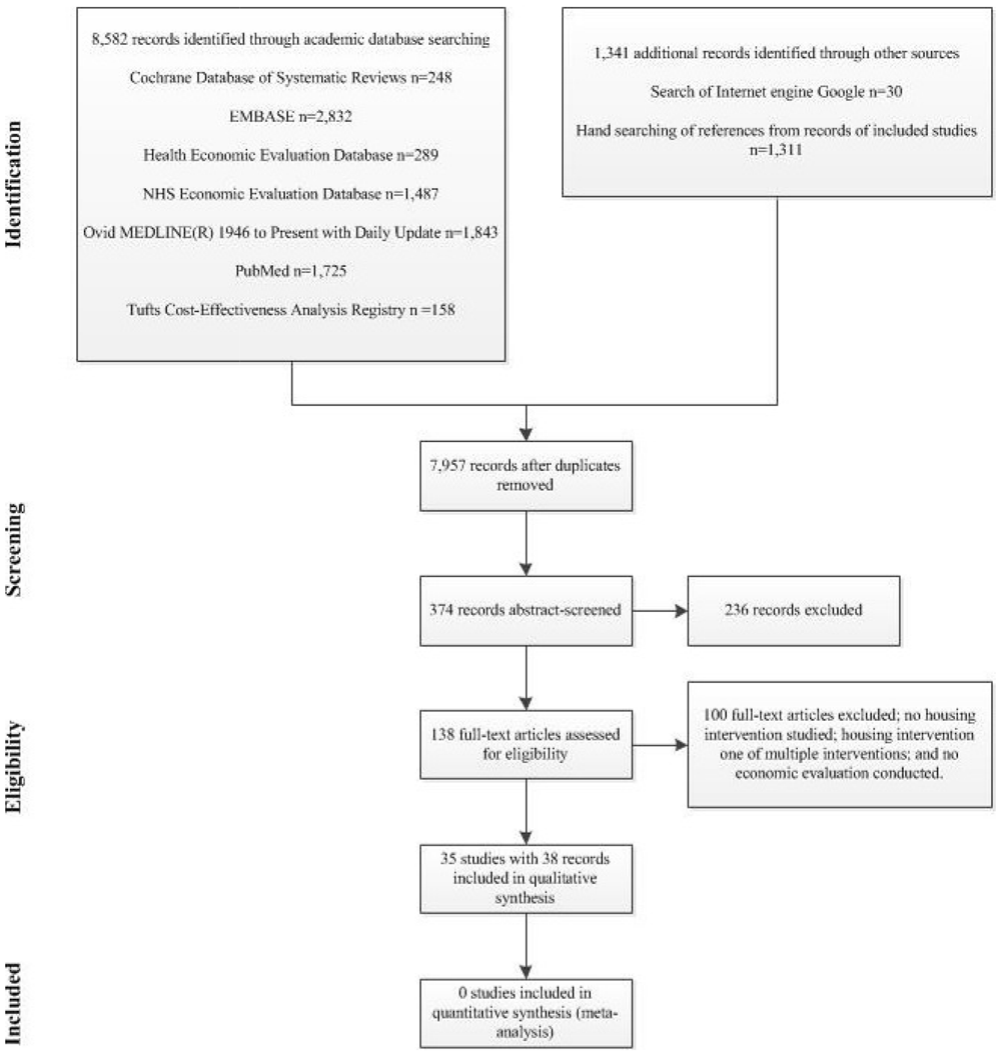
Flow-chart of the selection of studies of economic analyses of housing interventions.

The search identified a total of 7,957 records (without duplicates). Fig 2 presents a PRISMA flow-chart of the selection of studies. After title screening, 374 records were considered potentially relevant. After abstract screening, 138 records of 135 studies were still considered potentially relevant and these progressed to in-depth full-text screening. This next screening phase identified 34 studies with 37 records (described below). Hand searching of the reference lists of relevant previous systematic reviews [8, 18] identified one additional eligible study (Grimes et al 2012) [25]. The review included a final total of 35 studies with 38 records that fulfilled eligibility criteria, and they were included in the review and synthesized qualitatively. Study heterogeneity prohibited combining studies in meta-analysis.

### Study characteristics

Tables 1 and 2 present the characteristics of the studies included in this systematic review. Of the total of 35 included studies, 19 studies were cost-effectiveness analyses, 13 were cost-benefit analyses, one was a cost-utility analysis and two combined cost-effectiveness with cost-benefit analyses. The base year of cost data ranged between 1990 and 2014, although it was unclear in four studies. Most analyses used decision analytic modeling (n = 14 studies) or were conducted alongside randomized controlled trials or other experimental studies (n = 12), but some were conducted alongside observational studies (7), and two were conducted alongside intervention implementation. Slightly more studies took a health system perspective (22) than a societal perspective (13). Societal perspectives, in addition to health, covered one or more of the domains of climate change, energy, crime, education, income, productivity and wealth. The time horizon was short (i.e., 1–4 years) in 14 studies, medium (i.e., 5–10 years) in six studies, long (i.e., >10 years) in 11 studies and unclear in four studies.

**Table 1:** Characteristics of included studies, ordered by type of intervention.

Study	Setting	Base year of cost data	Study population	Intervention	Economic analysis type	Study design	Perspective	Time horizon
*Provided insecticide-treated bednets (ITBNs) or insecticide-treated hammocks (ITHs)*								
Bhatia et al, 2004 [26]	Surat, Gujarat, India	1997	General population (all ages)	Provided ITBN	CEA	Experimental	Health system	Unclear
Goodman et al, 2001 [27]	KwaZulu-Natal, South Africa	1999	General population (all ages)	Provided ITBN	CEA	Experimental	Health system	1 year
Guyatt et al, 2002 [28, 29]	Gucha and Kisii, Kenya	2000	General population (all ages)	Provided ITBN	CEA	Observational	Health system	1 year
Kamolratanakul et al, 2001 [30]	Mae-Ramad, Tak, Thailand	1993	General population (all ages)	Provided ITBN	CEA	Experimental	Health system	1 year
Morel et al, 2005 [31]	Predominantly Western African region ^a^; predominantly Southern and Eastern African region ^b^	2000	General population (all ages)	Provided ITBN	CEA	Model based	Health system	10 years
Morel et al, 2013 [32]	Ninh Thuan, Vietnam	2012	General population (all ages)	Provided ITHs	CEA	Experimental	Societal	Unclear
Mueller et al, 2008 [33]	Togo	2004	Children (9–59 months)	Provided ITBN	CEA	Model based	Health system	3 years
Pulkki-Brännström et al, 2012[34]	Unspecified	2009	General population (all ages); children (0–4 years)	Provided ITBN	CEA	Model based	Health system	10 years
Smithuis et al, 2013[35]	Rakhine State, Myanmar	2013	Children (0–10 years)	Provided ITBN	CEA	Experimental	Health system	~10 months
Wiseman et al, 2003 [36]	Asembo and Gem, Kenya	1996	Children (0–4 years)	Provided ITBN	CEA	Experimental	Health system	1 year
Yukich et al, 2008 [37]	Eritrea; Malawi; Senegal; Tanzania; Togo	2005	General population (all ages); children (0–4 years)	Provided ITBN	CEA	Model based	Health system	Up to 5 years
Yukich et al, 2009 [38]	Eritrea	2005	Children (unspecified age)	Provided ITBN	CEA	Alongside intervention implementation	Health system	5 years
*Provided home safety assessment and modification (HSAM)*								
Church et al, 2011 [39]	Australia	Unclear	Older people (≥ 65 years); older people (≥ 65 years) with high fall risk	Provided HSAM	CEA	Model based	Health system	35 years
Frick et al, 2010 [40]	US	Unclear	Older people (≥ 65 years) with high fall risk	Provided HSAM	CUA	Model based	Health system	Unclear
Jutkowitz et al, 2012 [41]	US	2003	Older people (≥ 70 years)	Provided HSAM	CEA	Model based	Health system	2 years
Keall et al, 2014[42]	New Zealand	2014	People in households residing in an owner-occupied dwelling constructed before 1980 and with one or more members receiving a state benefit or subsidy	Provided HSAM	CBA	Experimental	Health system	20 years
Kochera et al, 2002 [43]	US	2000	Older people (≥ 65 years)	Provided HSAM	CBA	Model based	Health system	1 year
Ling et al, 2008 [44]	Hanna, Maui, Hawaii, US	Unclear	Older people (≥ 65 years) with high fall risk	Provided HSAM	CBA	Observational	Health system	1 year
Salkeld et al, 2000 [45]	Part of Central Sydney Area, Australia	1997	Older people (≥ 65 years)	Provided HSAM	CEA	Experimental	Health system	1 year
*Ventilated home*								
Franchimon et al, 2008 [46]	Netherlands	2003	General population (all ages)	Ventilated home (dwellings only), ventilated home (dwellings, schools, offices)	CEA	Model based	Health system	Entire life span of the Dutch population in 2003
*Removed indoor lead (paint and dust)*								
Brown, 2002 [47]	US	2001	Children (unspecified age)	Removed indoor lead (public policy enforcement)	CBA	Model based	Societal (health, education, income)	Up to lifetime for data inputs
Dixon et al, 2012 [48]	Burlington, Bennington, Springfield, and scattered locations, Vermont; Minneapolis, St Paul, and Duluth, Minnesota; Cleveland and Chicago, US	Unclear	Children (unspecified age)	Removed indoor lead	CBA	Observational	Societal (health, education, energy, wealth)	Up to lifetime for data inputs
Gould, 2009 [49]	US	2006	Children (1–5 years)	Removed indoor lead	CBA	Model-based	Societal (health, education, crime, wealth)	Up to lifetime for data inputs
Nevin et al, 2008 [50]	US	2005	Children (1–5 years)	Removed indoor lead (replaced lead-unsafe with lead-safe windows)	CBA	Observational	Societal (health, wealth, energy)	Up to lifetime for data inputs
Pichery et al, 2011 [51]	France	2008	Children (1–6 years)	Removed indoor lead (lead-paint abatement)	CBA	Observational	Societal (health, education, crime)	Up to lifetime for data inputs
*Retrofitted insulation and/or installed heaters*								
Barton et al, 2007[10]	UK	2000	General population living in deprived geographic areas (all ages)	Retrofitted insulation; installed heaters	CEA	Experimental	Societal (health, education, energy)	1 year
Grimes et al, 2012 [25]	New Zealand	2009	General population (all ages) on low- or middle-income and living in houses built before 2000	Retrofitted insulation; installed heaters	CBA	Observational	Societal (health, energy)	30 years for retrofitting insulation, 10 years for installing heaters
Chapman et al, 2004 [11–13]	New Zealand	2002	General population (all ages)	Retrofitted insulation	CBA	Experimental	Societal (health, energy, environment)	30 years
Levy et al, 2003 [52]	US	1990	Families in single-family homes	Retrofitted insulation	CBA	Model based	Societal (health, productivity, energy)	1 year
Preval et al, 2010 [53]	New Zealand	2007	Households using either an unflued gas or electric plug-in heater as the main form of heating and with one or more children (7–12 years) with doctor-diagnosed asthma who had asthma symptoms in the last 12 months	Installed clean heater (heat pump, pellet burner, flued gas heater)	CBA	Observational	Societal (health, energy, climate)	12 years
*Gave away and/or installed smoke alarms*								
Ginnelly et al, 2005 [54]	Camden and Islington, London, UK	1999	General population (all ages)	Gave way smoke alarms	CEA	Experimental	Societal (health, wealth)	2 year
Haddix et al, 2001 [55]	Oklahoma City, Oklahoma, US	1990	General population (all ages)	Gave way smoke alarms	CBA	Alongside intervention implementation	Health system, societal (health, productivity)	5 years
Liu et al, 2012	US	2011	General population living in small communities of ≤5,000 population (all ages)	Gave way smoke alarms, installed smoke alarms	CEA, CBA	Model based	Societal (health, productivity, wealth)	1 year
*Regulated tap water*								
Han et al, 2007 [56]	Ontario, Canada	2002	Children (0–9 years)	Regulated tap water	CEA	Model based	Health system	10 years
Phillips et al, 2011 [57]	UK	2008	Children (0–4 years)	Regulated tap water	CEA, CBA	Experimental	Health system	Unclear

**Table 2:** Characteristics of included studies, ordered by type of intervention (Table 1 continued).

Effectiveness outcome measure (health-related)	Source of estimation of effectiveness	Source of estimation of resource utilization	Discount rates used	Sensitivity analysis for assumptions presented	Equity analysis presented	Comments
Other outcome (malaria infection averted)	Secondary data (records)	Secondary data (records)	Unclear discounting on effects, cost discounted by different, not specified percentages	Yes	No	
Life saved	Secondary data (records)	Secondary data (records)	None on effects, 5.3% on (selected) costs	Yes	No	
Other outcomes (malaria vector exposure averted, malaria infection averted)	Primary data (survey)	Secondary data (records)	None on effects, 3% on (selected) costs	Yes	No	
Other outcome (malaria infection averted)	Secondary data (records)	Secondary data (records)	None on effects or costs	No	No	
DALYs averted	Literature, secondary data (records), expert opinion	Literature, secondary data (records), expert opinion	Unclear	Yes	No	
Other outcome (malaria infection averted)	Primary data (survey), secondary data (trial)	Primary data (survey), secondary data (trial)	None on effects, 3% on costs	Yes	No	Not in prior SRs [8, 18].
DALYs averted, life saved, other outcome (malaria infection averted)	Literature	Primary data (survey)	3% on effects, 5% on costs	Yes	No	Not in prior SRs [8, 18].
DALYs averted	Primary data (from RCT)	Literature, secondary data (records)	Unclear	No	No	Not in prior SRs [8, 18].
DALYs averted, life saved	Literature	Literature	3% on effects and costs	Yes	Yes (general population vs children)	Not in prior SRs [8, 18].
LYS, life saved, other outcome (all cause sick child visit averted)	Secondary data (records, from trial)	Literature, secondary data (records), primary data (survey)	3% on effects and costs	Yes	No	Not in prior SRs [8, 18].
DALYs averted, death averted, other outcome (person year protected from malaria infection)	Secondary data (records)	Literature, secondary data (records)	3% on effects and costs	Yes	No	
DALYs averted, life saved	Literature	Secondary data (records)	3% on effects and costs	Yes	No	Intervention also provided in ante-natal care facilities. Not in prior SRs [8, 18].
QALYs gained	Literature	Literature	5% on effects and costs	Yes	No	Not in prior SRs [8, 18].
QALYs gained	Literature	Literature	3% on effects and costs	Yes	No	Not in prior SRs [8, 18].
LYS	Literature	Literature	3% on effects, none of costs	Yes	No	Intervention also included exercise training. Not in prior SRs [8, 18].
DALYs averted	Secondary data (from RCT)	Secondary data (from RCT)	3% on effects, none on costs	Yes	No	Not in prior SRs [8, 18].
Other outcome (fall averted)	Literature	Literature, expert opinion	Unclear	No	No	Not in prior SRs [8, 18].
Other outcome (fall averted)	Literature	Literature, field study	None on effects or costs	Yes	No	Not in prior SRs [8, 18].
Other outcome (fall averted)	Primary data (RCT)	Primary data (RCT)	None on effects or costs	Yes	Yes (all participants vs participants with a falls history)	Population included some people with cognitive impairments. Not in prior SRs [8, 18].
DALYs averted	Literature	Operational costs	None on effects or costs (depreciation of costs was estimated)	Yes	No	Intervention included installation of tobacco smoke, presence and humidity detectors. Intervention also provided in schools and offices. Potential conflict of interest, i.e. funded by a society encouraging technological, economical and scientific developments in the Dutch building services industry. Not in prior SRs [8, 18].
Other outcome (lead poisoning averted)	Literature	Literature	None on effects, 3% on costs	Yes	No	Not in prior SRs [8, 18].
Other outcome (lead poisoning averted)	Unclear	Unclear	None on effects or costs	No	No	Study did not consider costs associated with all health effects from lead exposure. Not in prior SRs [8, 18].
Other outcomes (lead poisoning averted, ADHD averted)	Literature	Literature	None on effects or costs	No	No	Not in prior SRs [8, 18].
Other outcomes (lead poisoning averted, ADHD averted, mental retardation averted)	Literature, primary data (survey)	Literature	None on costs, 3% on benefits.	No	No	Not in prior SRs [8, 18].
Other outcome (lead poisoning averted)	Literature	Literature	None on effects (except 3% on earnings) or costs (except 3% on abatement costs)	Yes	No	Not in prior SRs [8, 18].
Other outcome (psychological distress averted)	Secondary data (from RCT)	Literature, secondary data (from RCT)	None on effects, 3.5% on costs	Yes	No	Intervention also included: re-roofing, rewiring, ventilation systems and cavity wall.
Other outcomes (GP visit averted, hospitalization averted)	Secondary data (records)	Literature, secondary data (records)	5% on effects and costs	Yes	No	Majority of study population on low incomes.
Life saved, other outcomes (medical visit averted, hospitalization averted, medication averted)	Secondary data (records)	Secondary data (records)	4% on effects and costs	Yes	Yes (low- and middle-income vs high-income population; cooler vs warmer geographic regions)	Not in prior SRs [8, 18].
Life saved, other outcome (asthma averted)	Primary data (survey)	Literature	5% on effects and costs	No	No	Not in prior SRs [8, 18].
Other outcomes (visit to health professional averted, medication averted)	Secondary data (from RCT)	Secondary data (from RCT)	5% on effects and costs	Yes	No	Intervention implemented in homes previously retrofitted with insulation. Not in prior SRs [8, 18].
Life saved, other outcome (injury)	Secondary data (records)	Secondary data (records)	Unclear	No	No	Study population resided in deprived geographic areas. Not in prior SRs [8, 18].
Life saved, other outcome (injury)	Secondary data (records)	Secondary data (records)	None on effects, 3% on costs	Yes	No	Not in prior SRs [8, 18].
QALYs gained, LYS	Literature	Literature	3% on effects and costs	Yes	No	Not in prior SRs [8, 18].
Other outcome (scald averted)	Secondary data (records)	Secondary data (records)	3% on effects and costs	Yes	No	Intervention modified home safety indirectly through legislation and included education. Not in prior SRs [8, 18].
Other outcome (scald averted)	Primary data (survey) (from trial)	Secondary data (records)	None on effects, 3.5% on (selected) costs	Yes	Yes (children in all vs in the most disadvantaged geographic areas)	Intervention included education. Participants resided in social housing. Not in prior SRs [8, 18].

Notes:

ADHD: attention deficit hyperactivity disorder; CBA: cost-benefit analysis; CEA: cost-effectiveness analysis; CUA: cost-utility analysis; QALY: quality-adjusted life-year; DALY: disability-adjusted life-year; HSAM: home safety assessment and modification; ITBN: insecticide-treated bednets; ITHs: insecticide-treated hammocks; LYS: life-years saved; SRs: systematic reviews; UK: United Kingdom; US: United States.

^a^ Algeria, Angola, Benin, Burkina Faso, Cameroon, Cape Verde, Chad, Comoros, Equatorial Guinea, Gabon, Gambia, Ghana, Guinea, Guinea Bissau, Liberia, Madagascar, Mali, Mauritania, Mauritius, Niger, Nigeria, Sao Tome and Principe, Senegal, Seychelles, Sierra Leone and Togo.

^b^ Botswana, Burundi, Central African Republic, Congo, Cote d’Ivoire, Democratic Republic of the Congo, Eritrea, Ethiopia, Kenya, Lesotho, Malawi, Mozambique, Namibia, Rwanda, South Africa, Swaziland, Uganda, United Republic of Tanzania, Zambia and Zimbabwe.

The most common country setting was the United States (n = 11 studies), followed by New Zealand (4) and the United Kingdom (3). Two studies each were conducted for Australia, Eritrea, Kenya and Togo, and one study each for: Canada, France, India, Malawi, Myanmar, the Netherlands, Senegal, South Africa, Tanzania, Thailand, Vietnam, the Southern / Eastern African region and the Western African region. Study populations were the community-dwelling, healthy general population (n = 12 studies), children (13), older people (6) and/or specific population groups (e.g., persons residing in geographically deprived areas or persons earning low or middle incomes) (6). Study populations were generally well-tailored to the studied interventions. For example, most HSAM interventions were studied in older people, and indoor lead paint removal was studied in children.

**Fig 3:**
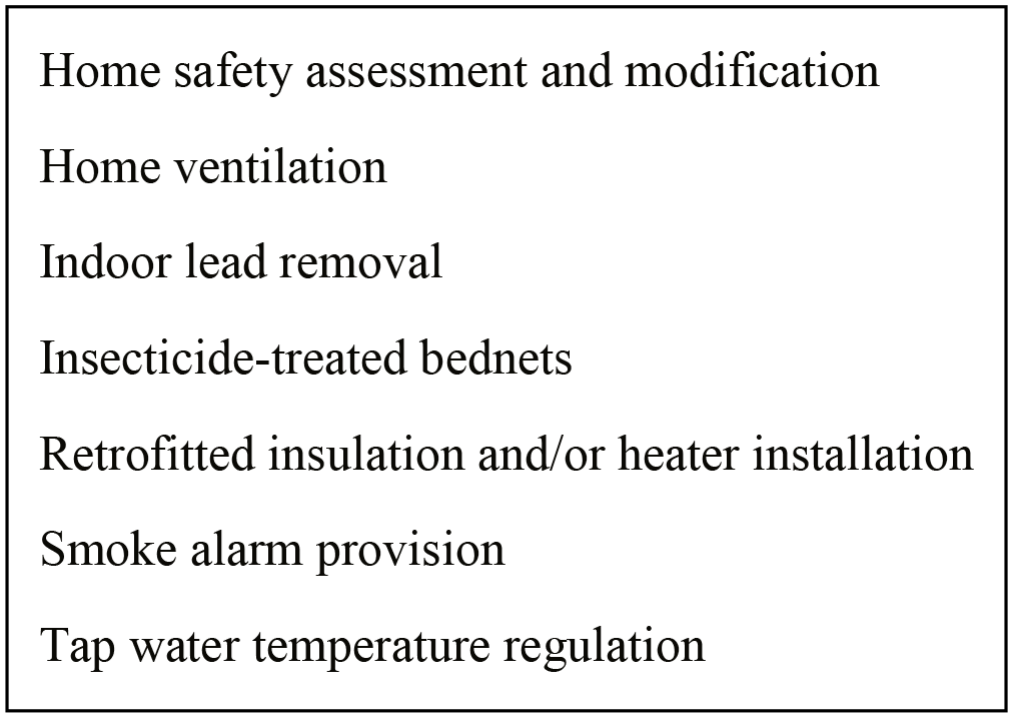
Intervention types included in the study.

This systematic review included six broad types of housing interventions (Fig 3) in addition to the provision of ITBNs or ITHs to prevent malaria or other infections. The latter were examined in 12 studies [26–36, 38]. These interventions differed in terms of the type of bednet or hammock (conventional versus long-lasting) and the type of insecticide used.

HSAM interventions were studied in seven studies [39–45]. All of these interventions aimed to reduce falls in older people (except one study that aimed to reduce injury in the general population [42]) by identifying and removing fall and other injury hazards in the home, for example by installing hand rails on stairs or in the bathroom and removing trip hazards. Removal of indoor lead was examined in five studies [47–51]. These interventions included removal of lead-containing window frames and other indoor materials, as well as enforcement of public policy for removing indoor lead. Retrofitting of insulation and/or installation of heaters was examined in five studies [10–13, 25, 52, 53]. Finally, giving away or installing smoke alarms was examined in three studies [54, 55, 58], regulating household tap water temperature to prevent scalds was examined in two studies [56, 57], and increasing home ventilation was examined in one study [46].

Effectiveness outcome measures were QALYs gained (n = 3 studies), DALYs averted (8), life-years saved (LYS) (2), lives saved or deaths averted (10) and/or one or more other outcomes (24 studies). Other outcomes comprised morbidity ones (e.g., asthma, attention deficit hyperactivity disorder, lead poisoning, malaria infection, mental retardation or psychological distress), injury (e.g., fall or scald), medication and use of health services (e.g., GP visit and hospitalization). Effectiveness estimates were sourced primarily from the literature (n = 14 studies) and secondary data (15), and less commonly from primary data (7), expert opinion and/or an unclear source (1 each). Estimates of resource utilization were sourced from secondary data (19), literature (18 studies), primary data (3), expert opinion (2), unclear sources (2) and/or a field study or operational costs (1 each). If discount rates were used, they ranged between 3% and 5.3%, but were most commonly either 3% or 5% and applied to both effects and costs. However, just under half of all included studies (17) did not discount either or both of effects and costs, and four studies did not discuss discounting. Sensitivity analyses were conducted in most (27) studies. Only four studies reported some form of equity analysis, which were generally limited to comparisons of targeted (to disadvantaged populations) and untargeted strategies. For example, one study compared the cost-benefit of retrofitting insulation targeted to the low- and middle-income populations with that of the intervention targeted to the high-income population [11–13].

### Quality assessment

Table 3 presents our quality assessments of the included studies, excluding non-applicable items and using percentage scores as indicators. The quality varied across included studies, ranging in average score from 33% to 98% (median: 72%). The studies with the highest average scores were Liu et al 2012 (98%) [58], Bhatia et al 2004 (91%) [26] and Preval et al 2010 (83%) [53]. For interventions examined by four or more studies, studies on provision of smoke alarms received the highest average score (83%), followed by studies on provision of ITBNs or ITHs (73%) and retrofitting insulation and/or installing heaters (68%). Relatively low average scores were received by studies of removal of indoor lead (63%) and HSAM (62%). On average, studies scored highly on the categories of effectiveness estimation (94% of the maximum attainable score over all studies) and interpretation of results (86%). However, cost estimation, study design and analysis scored relatively poorly with 54%, 59% and 62%, respectively. We found the study [46] on home ventilation for preventing chronic lung disease to have a potential financial conflict of interest, because it received funding from “a society that aims to encourage technical, economical and scientific developments in the Dutch building services industry” (p. 384). The range over which the cost-effectiveness estimates ranged appeared plausible and study authors’ conclusions included “not cost-effective” in some studies, which provided no evidence for publication bias.

**Table 3:** Summary of quality assessment and domain scores of included studies.

Authors		Scored domains					Summary scores		
		Study design	Effectiveness estimation	Cost estimation	Analysis	Interpretation of results	Number of items scored	Sum of scores	Total average score
*Provided insecticide-treated bednets or insecticide-treated hammocks*									
Bhatia et al, 2004 [26]	Score granted	11	8	7	17	10	29	53	1.83
	% of maximum (domain) score	79%	100%	88%	94%	100%			91%
Goodman et al, 2001 [27]	Score granted	12	8	3	14	10	30	47	1.57
	% of maximum (domain) score	86%	80%	38%	78%	100%			78%
Guyatt et al, 2002 [28, 29]	Score granted	9	8	1	3	9	26	30	1.15
	% of maximum (domain) score	64%	100%	17%	21%	90%			58%
Kamolratanakul et al, 2001 [30]	Score granted	11	8	2	9	9	27	39	1.44
	% of maximum (domain) score	79%	100%	25%	64%	90%			72%
Morel et al, 2005 [31]	Score granted	10	6	3	8	10	28	37	1.32
	% of maximum (domain) score	71%	75%	38%	50%	100%			66%
Morel et al, 2013 [32]	Score granted	9	8	6	7	10	28	40	1.43
	% of maximum (domain) score	64%	100%	75%	44%	100%			71%
Mueller et al, 2008 [33]	Score granted	8	10	6	15	9	30	48	1.60
	% of maximum (domain) score	57%	100%	75%	83%	90%			80%
Pulkki-Brännström et al, 2012 [34]	Score granted	11	7	4	13	10	28	45	1.61
	% of maximum (domain) score	79%	88%	50%	81%	100%			80%
Smithuis et al, 2013 [35]	Score granted	8	8	2	7	9	28	34	1.21
	% of maximum (domain) score	57%	100%	25%	44%	90%			61%
Wiseman et al, 2003 [36]	Score granted	8	7	7	9	9	28	40	1.43
	% of maximum (domain) score	57%	88%	88%	56%	90%			71%
Yukich et al, 2008 [37]	Score granted	10	7	5	8	9	28	39	1.39
	% of maximum (domain) score	71%	88%	63%	50%	90%			70%
Yukich et al, 2009 [38]	Score granted	7	10	6	10	9	29	42	1.45
	% of maximum (domain) score	50%	100%	75%	63%	90%			72%
*Provided home safety assessment and modification*									
Church et al, 2011 [39]	Score granted	8	8	1	17	10	28	44	1.57
	% of maximum (domain) score	57%	100%	17%	94%	100%			79%
Frick et al, 2010 [40]	Score granted	11	8	3	16	9	29	47	1.62
	% of maximum (domain) score	79%	100%	38%	89%	90%			81%
Jutkowitz et al, 2012 [41]	Score granted	6	8	3	16	10	29	43	1.48
	% of maximum (domain) score	43%	100%	38%	89%	100%			74%
Keall et al, 2014 [42]	Score granted	6	6	6	6	6	28	30	1.07
	% of maximum (domain) score	43%	75%	75%	38%	60%			54%
Kochera et al, 2002 [43]	Score granted	5	5	4	5	2	28	21	0.75
	% of maximum (domain) score	36%	63%	50%	31%	20%			38%
Ling et al, 2008 [44]	Score granted	4	7	2	2	3	27	18	0.67
	% of maximum (domain) score	29%	88%	25%	14%	30%			33%
Salkeld et al, 2000 [45]	Score granted	7	8	6	11	9	28	41	1.46
	% of maximum (domain) score	50%	100%	75%	69%	90%			73%
*Increased home ventilation*									
Franchimon et al, 2008 [46]	Score granted	7	7	3	9	6	28	32	1.14
	% of maximum (domain) score	50%	88%	38%	56%	60%			57%
*Removed indoor lead (paint and dust)*									
Brown, 2002 [47]	Score granted	11	8	5	14	8	29	46	1.59
	% of maximum (domain) score	79%	100%	63%	78%	80%			79%
Dixon et al, 2012 [48]	Score granted	6	6	2	3	9	27	26	0.96
	% of maximum (domain) score	43%	75%	25%	21%	90%			48%
Gould, 2009 [49]	Score granted	5	6	4	4	9	27	28	1.04
	% of maximum (domain) score	36%	75%	50%	29%	90%			52%
Nevin et al, 2008 [50]	Score granted	5	7	4	6	8	27	30	1.11
	% of maximum (domain) score	36%	88%	50%	43%	80%			56%
Pichery et al, 2011 [51]	Score granted	11	7	8	10	10	28	46	1.64
	% of maximum (domain) score	79%	88%	100%	63%	100%			82%
*Retrofitted insulation and/or installed heaters*									
Barton et al, 2007 [10]	Score granted	3	6	6	13	4	29	32	1.10
	% of maximum (domain) score	21%	75%	75%	72%	40%			55%
Chapman et al, 2004 [11–13]	Score granted	7	8	3	11	10	27	39	1.44
	% of maximum (domain) score	50%	100%	38%	79%	100%			72%
Grimes et al, 2012 [25]	Score granted	8	7	6	10	8	28	39	1.39
	% of maximum (domain) score	57%	88%	75%	63%	80%			70%
Levy et al, 2003 [52]	Score granted	5	8	3	8	9	28	33	1.18
	% of maximum (domain) score	36%	100%	38%	50%	90%			59%
Preval et al, 2010 [53]	Score granted	13	8	5	12	10	29	48	1.66
	% of maximum (domain) score	93%	100%	63%	67%	100%			83%
*Provided smoke alarm*									
Ginnelly et al, 2005 [54]	Score granted	7	7	5	6	9	23	34	1.48
	% of maximum (domain) score	50%	88%	83%	75%	90%			74%
Haddix et al, 2001 [55]	Score granted	9	8	6	9	10	27	42	1.56
	% of maximum (domain) score	64%	100%	75%	64%	100%			78%
Liu et al, 2012 [58]	Score granted	14	10	7	18	10	30	59	1.97
	% of maximum (domain) score	100%	100%	88%	100%	100%			98%
*Regulated tap water temperature*									
Han et al, 2007 [56]	Score granted	8	7	1	11	9	27	36	1.33
	% of maximum (domain) score	57%	70%	17%	79%	90%			67%
Phillips et al, 2011 [57]	Score granted	10	8	4	8	10	27	40	1.48
	% of maximum (domain) score	71%	100%	50%	57%	100%			74%
Total average domain score (%)		59%	94%	54%	62%	86%			

### Study findings

Table 4 presents study findings. Overall, there was a considerable body of evidence on the cost-effectiveness of the health impact of several housing interventions (including for ITBNs) in several populations and country settings. In general, this body of evidence suggested that these interventions were moderately to highly cost-effective or cost-beneficial, although cost-effectiveness varied by different intervention types.

**Table 4:** Interventions compared, study objectives and main study conclusions of included studies.

Authors	Comparison	Study objective	Study authors’ health-related conclusions [Reviewers’ health-related conclusions]
*Provided insecticide-treated bednets (ITBNs) or insecticide-treated hammocks (ITHs)*			
Bhatia et al, 2004 [26]	ITBNs vs standard practice	To estimate the cost-effectiveness of ITBNs in preventing malaria infection	ITBNs had a cost per malaria infection averted of Rs1,848 (US$52). [Assuming a cost-effectiveness threshold of US$50 per malaria infection averted, ITBNs were not cost-effective.]
Goodman et al, 2001 [27]	ITBNs vs indoor residual spraying	To estimate the cost-effectiveness of ITBNs in preventing malaria infection	ITBNs were more effective and more costly at a cost per death averted of R11,718 (US$1,915).
Guyatt et al, 2002 [28, 29]	ITBNs vs standard practice	To estimate the cost-effectiveness of ITBNs in preventing malaria infection [28]	ITBNs had a cost per malaria infection averted of US$29 [28]. [Assuming a cost-effectiveness threshold of US$50 per malaria infection averted, ITBNs were highly cost-effective.]
As above	As above	To estimate the cost-benefit of ITBNs in preventing malaria infection [29]	ITBNs had a cost per person protected from malaria infection of US$2.34 [29]. [Assuming a cos-effectiveness threshold of US$50 per malaria infection averted, ITBNs were highly cost-effective.]
Kamolratanakul et al, 2001 [30]	ITBNs vs standard practice	To estimate the cost-effectiveness of ITBNs in preventing malaria infection	ITBNs had a cost per malaria infection averted of US$1.54. [Assuming a cost-effectiveness threshold of US$50 per malaria infection averted, ITBNs were highly cost-effective.]
Morel et al, 2005 [31]	ITBNs plus other interventions^a^ vs standard practice	To estimate the cost-effectiveness of ITBNs plus other interventions^a^ in preventing malaria infection	ITBNs plus other interventions^a^ were cost-effective at incremental cost per DALY averted of at less than Int$60 in both regions of Africa.
Morel et al, 2013 [32]	Long-lasting ITHs vs standard practice	To estimate the cost-effectiveness of using long-lasting ITHs in preventing malaria infection	Long-lasting ITHs could be cost-effective at average savings per malaria infection averted of US$14.60. [Assuming a cost-effectiveness threshold of US$50 per malaria infection averted, long-lasting ITHs were highly cost-effective.]
Mueller et al, 2008 [33]	Long-lasting ITBNs vs standard practice	To estimate the cost-effectiveness of ITBNs in preventing malaria infection among children	Long-lasting ITBNs were cost-effective at a cost per DALY averted of US$16.39.
Pulkki-Brännström et al, 2012 [34]	Long-lasting ITBNs vs conventional ITBNs	To estimate the cost-effectiveness of long-lasting ITBNs compared with conventional ITBNs in preventing malaria infection	If conventional and long-lasting ITBNs have the same physical lifespan (3 years), long-lasting ITBNs are more cost-effective unless they are priced at more than US$1.5 above the price of conventional nets. Distributing replenishment nets each year in addition to the replacement of all nets every 3–4 years costs US$1,080 to US$1,610 per additional under-5 death averted.
Smithuis et al, 2013 [35]	ITBNs vs standard practice	To estimate the cost-effectiveness of ITBNs in preventing malaria infection among children	ITBNs had a cost per DALY averted of Int$51. [It is impossible to contextualize this in terms of the per capita GDP as this measure is not available in 2013 for Myanmar [24], but we consider the cost per DALY averted to be highly cost-effective.]
Wiseman et al, 2003 [36]	ITBNs vs standard practice	To estimate the cost-effectiveness of ITBNs in preventing malaria infection among children	ITBNs were highly cost-effective at a cost per LYS of US$34.
Yukich et al, 2008 [37]	Long-lasting ITBNs vs conventional ITBNs	To estimate the cost-effectiveness of long-lasting ITBNs vs conventional ITBNs in preventing malaria infection	Long-lasting ITBNs were more cost-effective than conventional ITBNs. The cost per treated-net year of protection ranged from US$1.38 in Eritrea to US$1.90 in Togo for long-lasting ITBNs, but from US$1.21 in Eritrea to US$6.05 in Senegal for conventional ITBNs.
		To estimate the cost-effectiveness of long-lasting ITBNs vs conventional ITBNs in preventing malaria infection among children	Long-lasting ITBNs were more cost-effective than conventional ITBNs among children. The cost per child death averted ranged from US$502 to US$692 for long-lasting ITBNs, but from US$438 to US$2,199 for conventional ITBNs.
Yukich et al, 2009 [38]	ITBNs vs standard practice	To estimate the cost-effectiveness of ITBNs in preventing malaria infection among children	ITBNs were cost-effective at a cost per DALY averted of US$13 to US$44.
*Provided home safety assessment and modification (HSAM)*			
Church et al, 2011 [39]	HSAM vs standard practice	To estimate the cost-effectiveness of HSAM in preventing falls among older people with a previous injurious fall	HSAM had a cost per QALY gained of AU$57,856. [Assuming a base year of 2010 and one 2010 per-capita GDP of Australia [24] as the cost-effectiveness threshold [23], HSAM was highly cost-effective.]
Frick et al, 2010 [40]	HSAM vs standard practice	To estimate the cost-effectiveness of HSAM in preventing falls among older people	HSAM was less expensive and more effective than standard of care.
Jutkowitz et al, 2012 [41]	HSAM vs standard practice	To estimate the cost-effectiveness of HSAM in addressing functional difficulties, performance goals and home safety among older people	HSAM had a cost per LYS of US$13,179. Investment in HSAM may be worthwhile depending on society’s willingness to pay.
Keall et al, 2014 [42]	HSAM vs standard practice	To estimate the cost-effectiveness of HSAM in preventing injuries among the general population	HSAM was very cost-effective at a cost per DALY averted of NZ$14,300.
Kochera et al, 2002 [43]	HSAM vs standard practice	To estimate the cost-benefit of HSAM in preventing falls among older people	HSAM had a cost per injury averted of US$8,319.
Ling et al, 2008 [44]	HSAM vs standard practice	To estimate the cost-benefit of HSAM in preventing falls among older people	HSAM was highly cost-saving at an average cost of US$800 and an averted cost of $1,728, with a cost-benefit ratio of 1:3.2.
Salkeld et al, 2000 [45]	HSAM vs standard practice	To estimate the cost-effectiveness of HSAM in preventing falls among older people and among older people with a previous injurious fall	HSAM had an average cost of AUS$1,921 per fall averted among all older people, but was cost-saving among older people with a previous injurious fall.
*Increased home ventilation*			
Franchimon et al, 2008 [46]	Increased home ventilation vs standard practice	To estimate the cost-effectiveness of building ventilation for preventing chronic lung disease	Increased home ventilation was cost-effective at a cost per DALY averted of €18,000.
*Removed indoor lead (paint and dust)*			
Brown, 2002 [47]	Strict versus limited enforcement of lead poisoning prevention housing policies	To estimate the cost-benefit of strict versus limited enforcement of lead poisoning prevention housing policies in preventing lead poisoning among children	Strict enforcement compared with limited enforcement had net benefits per lead poisoning averted of US$45,360.
Dixon et al, 2012 [48]	Lead-safe window replacement vs window repair	To estimate the cost-benefit of lead-safe window replacement in preventing lead poisoning among children	Lead-safe window replacement compared to window repair had net benefits of US$1,700 to US$2,000 per housing unit.
Gould, 2009 [49]	Lead abatement vs standard practice	To estimate the cost-benefit of lead abatement among children	Lead abatement had a large net benefit of $181 billion to US$269 billion, with a cost-benefit ratio of 1:17 to 1:221.
Nevin et al, 2008 [50]	Lead-safe window replacement vs standard practice	To estimate the cost-benefit of lead-safe window replacement in preventing lead poisoning among children	Lead-safe window replacement had a large net societal benefit of at least US$67 billion (including the benefit from preventing IQ reduction but excluding other health benefits).
Pichery et al, 2011 [51]	Lead abatement vs standard practice	To estimate the cost-benefit of lead abatement in preventing lead poisoning among children	Lead abatement had a large net benefit of €0.25 billion to €3.78 billion.
*Retrofitted insulation and/or installed heater*			
Barton et al, 2007 [10]	Retrofitting insulation and installing heater vs standard practice	To estimate the cost-effectiveness of retrofitting insulation and installing heaters	The study could not estimate the cost-effectiveness of retrofitting insulation and installing heater.
Chapman et al, 2004 [11–13]	Retrofitted insulation vs standard practice	To estimate the cost-benefit of retrofitting insulation	Retrofitting insulation was cost-beneficial, with a cost-benefit ratio of 1:1.5 to 1:2.
Grimes et al, 2012 [25]	Retrofitted insulation and installed heater vs standard practice	To estimate the cost-benefit of retrofitting insulation and installing heaters	Retrofitting insulation and installing heaters had a net benefit of NZ$0.95 billion. The benefits attributable to retrofitting insulation dominated, and the study could not estimate the benefits attributable to installing heaters.
Levy et al, 2003 [52]	Retrofitted insulation vs standard practice	To estimate the cost-benefit of retrofitting insulation	Retrofitting insulation averted health costs of US$1.3 billion per year.
Preval et al, 2010 [53]	Targeted installing of heater vs standard practice	To estimate the cost-benefit of targeted installing of heaters	Targeted installing of heaters was cost saving from health-related benefits alone, with a cost-benefit ratio of 1:1.09.
	Untargeted installing of heaters vs standard practice	To estimate the cost-benefit of untargeted installing of heaters	Untargeted installing of heaters was not cost saving, with a cost-benefit ratio of 1:0.31.
*Provided smoke alarms*			
Ginnelly et al, 2005 [54]	Gave away smoke alarms vs standard practice	To estimate the cost-effectiveness of giving away smoke alarms for reducing fire-related injury and death	Giving away smoke alarms is unlikely to be cost-effective.
Haddix et al, 2001 [55]	Gave away smoke alarms vs standard practice	To estimate the cost-effectiveness of giving away smoke alarms for reducing fire-related injury and death	Giving away smoke alarms was cost saving, with almost US$1 million saved over five years.
Liu et al, 2012 [58]	Gave away smoke alarms vs standard practice	To estimate the cost-effectiveness of giving away and installing smoke alarms for reducing fire-related injury and death	Installing smoke alarms was more cost-effective than giving away smoke alarms at an average cost-effectiveness ratio per QALY gained of US$51,404 and US$45,630, respectively.
	Installed smoke alarm vs standard practice	To estimate the cost-benefit of installing smoke alarms for reducing fire-related injury and death	Both giving away and installing smoke alarms were cost-beneficial, with cost-benefit ratios of 1:2.1 and 1:2.3, respectively.
*Regulated tap water temperature*			
Han et al, 2007 [56]	public health legislative / educational strategy vs standard practice	To estimate the cost-effectiveness of a public health legislative / educational strategy for reducing tap water scalds among children	A public health legislative / educational strategy was cost saving, with a cost per scald averted of C$531.
Phillips et al, 2011 [57]	Installing thermostatic mixer valves vs standard practice	To estimate the cost-effectiveness of installing thermostatic mixer valves for reducing tap water scalds	Installing thermostatic mixer valves is very likely to be cost-effective at the cost per bath water scald averted of £1,887 to £75,520.
		To estimate the cost-benefit of installing thermostatic mixer valves for reducing tap water scalds	Installing thermostatic mixer valves is very likely to be cost-beneficial, with a cost-benefit ratio of 1:1.4.

Notes:

AU$: Australian dollar; C$: Canadian dollar; DALY: disability adjusted life year; GDP: gross domestic product; HSAM: home safety assessment and modification; Int$: international dollar; ITBNs: insecticide-treated bednets; ITHs: insecticide-treated hammocks; LYS: life years saved; NZ$: New Zealand dollar; QALY: quality-adjusted life year; R: South African rand; Rs: Indian rupee; US$: United States dollar.

^a^ Case management with artemisinin based combination therapy plus intermittent presumptive treatment in pregnancy.

There was fairly consistent evidence for the cost-effectiveness or favorable cost-benefit of ITBNs or ITHs, indoor lead removal and retrofitting of insulation. The 12 studies of ITBNs or ITHs for preventing malaria or other insect-borne infections found these interventions to be moderately to highly cost-effective [26–36, 38]. This body of evidence included two studies [28–30] which found ITBNs to have an cost per malaria infection averted of less than US$50, and one study [35] which found ITBNs to have a cost per DALY averted of Int$51, which we regard to indicate high cost-effectiveness. The only exception was one study [26] that found ITBNs to have an cost per malaria infection averted of US$52, which could be of borderline cost-effectiveness assuming a cost-effectiveness threshold of US$50. Two studies found that, under certain conditions, long-lasting ITBNs were relatively more cost-effective than conventional ITBNs [34, 37].

Five studies on indoor lead removal for reducing lead poisoning and sequelae consistently found this intervention to be highly cost-beneficial, accruing considerable net benefits [47–51]. Three studies on retrofitting insulation found that retrofitting insulation was highly cost-effective or cost-beneficial [11–13, 25, 52].

The evidence was more mixed around the cost-effectiveness or cost-benefit for HSAM and provision of smoke-alarms. Seven studies of HSAM for reducing injury (predominantly falls) had mixed results, but tended towards being cost-effective/cost-beneficial overall. One study found HSAM not to be cost-effective among older people [43]. Another study found HSAM likely be cost-effective among older people [41]. Relatively high cost-effectiveness was found in two studies [40, 44] for older people and one study [42] for the general population. Similarly, another study [39] suggested HSAM was a very cost-effective intervention among older people, producing health gain below the WHO’s standard cost-effectiveness threshold [23] of the per-capita GDP level. Finally, one study found HSAM to be cost-saving among older people with a previous fall (but not among all older people) [45].

The three studies of smoke alarm provision found mixed evidence favoring cost-effectiveness. One study found that giving away smoke alarms was unlikely to be cost-effective [54]. In contrast, another found that both giving away and installing smoke-alarms was cost-effective and cost-beneficial, with the give-away programs being relatively more cost-effective than the installation program [58]. And the third study found this intervention to be cost-saving [55].

Finally, the economic evidence was inconclusive for installing heaters and insufficient for home ventilation and regulating tap water temperatures. Three studies of installing heaters could either not establish the cost-effectiveness of this intervention [10, 25] or found cost-effectiveness only if the intervention was targeted at households with high asthma rates [53]. One study found home ventilation to be cost-effective [46], but we note that this study may have had a financial conflict of interest. Both studies examining regulating tap water temperature found this intervention to be cost-effective [56, 57], but we judged this body of evidence too small in size to be considered conclusive.

## Discussion

### Main findings and interpretation

This review identified 35 economic analyses of housing improvement interventions, of which 12 involved ITBNs or ITHs. Most of the included studies were cost-effectiveness analyses that either used decision analytic modeling or were conducted alongside randomized controlled trials or other experimental studies and that adopted a health sector perspective. Overall, the quality of the body of evidence is probably acceptable for informing policy-making regarding some intervention types (i.e., provision of ITBNs or ITHs, lead paint removal and retrofitting of insulation). But there is still considerable scope for additional studies and for improvements in study quality for other types of housing interventions. Furthermore, few studies determined the relative cost-effectiveness of different housing interventions (i.e., HSAM versus home insulation), and no study determined the cumulative cost-effectiveness of implementing two or more housing interventions at once (e.g., HSAM plus home insulation).

In summary, this review found that several housing improvement interventions were cost-effective or cost-beneficial in general. There was a considerable amount of consistent evidence of relatively high quality for the cost-effectiveness or favorable cost-benefit of providing ITBNs or ITHs, removing indoor lead paint or dust and retrofitting insulation. In contrast, evidence on providing HSAM and giving away or installing smoke alarms while mostly of acceptable quality had mixed results (though favoring cost-effectiveness) and, for providing smoke alarms, was relatively small (n = 3 studies). The relatively small body of evidence on installing heaters was inconclusive, with two studies completely unable to estimate cost-effectiveness or cost-benefit. Finally, evidence on home ventilation and regulating tap water temperature was insufficient in size to judge value from a health economic perspective (n = 1 study and n = 2 studies, respectively) and, for home ventilation, was also of relatively low quality and with a potential financial conflict of interest.

The objective of this systematic review was to synthesize evidence on the cost-effectiveness of housing improvement interventions as policy tools for improving health and health equity, but the systematic review also found evidence that some housing interventions have considerable non-health co-benefits. For example, retrofitting insulation can cost-effectively improve health *and* at the same time also reduce domestic energy use and, in turn, anthropogenic greenhouse gas emissions [11–13]. Abating lead from the home can cost-effectively improve health over the life-course *and* can also result in savings from less special education, reduced crime and increased lifetime earnings and productivity [51]. Therefore, it could be argued that the economic benefits of housing interventions found in this review present an underestimate of their total benefits, and that the identified co-benefits further advance the economic case for the cost-effectiveness of housing interventions.

The only previous systematic review specifically of housing interventions covered only two studies [10–13] of two intervention types (installing heating and retrofitting insulation) in the community-dwelling, healthy population and concluded that there is a “near absence of economic evaluation of housing improvements” (p. 843) [8]. However, our updated review identified 23 housing improvement studies (in addition to the 12 on ITBNs) in community-dwelling, healthy populations. Similarly, we believe our review benefited from a broader scope for considering interventions.

Furthermore, in terms of ITBNs and ITHs, the most relevant previous systematic review [18] covered publications up to 2010, and included only seven studies [26, 27, 29–31, 36, 37]. However, our review identified 12 studies of this intervention type.

### Review strengths and weaknesses

As well as its relatively broad scope, a strength of this systematic review was that we searched several electronic databases, including three databases of health economic analyses. This helped ensure that we captured relevant economic analyses published in both the academic and grey literature. Also, the two review authors independently screened potentially relevant records for eligibility criteria, extracted data and assessed the quality of the included studies. As a result, we are reasonably confident that we have identified nearly all relevant studies in the academic literature from 1 January 2000 to 15 April 2014, but some relevant reports in the grey literature may have been missed.

### Potential implications for policy-making

The findings of this review provide support for the economic case [4] for addressing housing quality as a social determinant of health [1]. In particular, they help provide the economic case for interventions to remove lead paint and for retrofitting insulation.

In countries with malaria and donor countries supporting them, there is now even a stronger health economic case for the provision of ITBNs or ITHs. However, local circumstances (e.g., for ITBNs or ITHs, local levels of insecticide resistance and access to local malaria treatment services) will, of course, influence the cost-effectiveness of such interventions.

### Implications for future research and guideline revisions

The existing evidence is currently limited in its ability to determine whether housing improvement interventions are cost-effective policy tools for improving health equity. Only four out of the 35 included economic analyses considered health equity, and generally such considerations were limited to comparisons of the cost-effectiveness or cost-benefit of housing interventions when they were targeted versus untargeted to disadvantaged populations. This is therefore an important area for further research.

Future research should also address the currently mixed evidence (favoring cost-effectiveness) for providing HSAM and giving away or installing smoke alarms; inconclusive evidence for installing heaters; and insufficient evidence for improving home ventilation and regulating tap water. Existing evidence on most housing intervention types (except for providing ITBNs and ITHs) is currently limited to high-income countries (mostly only the United States, New Zealand and the United Kingdom).

For all housing intervention types, additional economic analyses of the health equity impact of housing interventions are required. Although best practice guidelines for economic analyses of interventions addressing the social determinants of health suggest that such analyses should consider health equity [4], this review found that few existing economic analyses of housing interventions have considered health equity even in a minor way. This leaves a considerable gap in the current body of evidence that future research should fill.

Moreover, a considerable number of studies included in the review took a relatively narrow health sector perspective, which is likely to underestimate the full economic benefits for society that housing interventions are likely to have. In line with WHO guidelines for economic analyses of interventions addressing the social determinants of health [4], future economic analyses of housing interventions should try to adopt a societal perspective and carefully include all relevant potential co-benefits, if feasible.

Furthermore, homes are particularly important sites of public health intervention, not only because most people spend considerable amounts of their time at home, but also because it is potentially possible to cost-effectively address multiple health hazards at once, potentially with multiple interventions. For example, a recently trialed HSAM intervention that comprehensively removed injury hazards (rather than only falls hazards) for all household members (rather than only older people in the household) has been found to be highly cost-effective [42]. Taking this one step further, future economic analyses should determine the cumulative economic value of combining several housing interventions. For example, the cost-effectiveness of combining the relatively broader HSAM intervention with other housing interventions such as insulation retrofitting is currently unknown.

Finally, this review also has methodological implications for systematically reviewing economic analyses of the health and health equity impact of interventions. A previous systematic review [8] of economic analyses with comparable focus (albeit smaller scope) that was conducted alongside a review of effectiveness identified two studies of community-dwelling, healthy populations, whereas our systematic review identified 23 relevant studies. This highlights the relative limitations of conducting systematic reviews of economic analyses alongside systematic reviews of effectiveness, if only the economic analyses conducted alongside studies included in the systematic review of effectiveness are included. This places emphasis on best practice guidelines [5] calling for comprehensive systematic reviews of economic analyses to include economic analyses using decision analytic modeling [7]. Furthermore, searching specifically for economic analyses and searching electronic databases of health economic analyses can considerably increase the coverage of systematic reviews of economic analyses of the health and health equity impact of interventions.

## Conclusions

This systematic review provides updated evidence that several housing improvement interventions (such as removing indoor lead and retrofitting insulation) and also the provision of insecticide-treated bednets are cost-effective interventions. Some of these interventions can also provide wider societal co-benefits (e.g., energy savings, greenhouse gas emission reductions or increased earnings). Nevertheless, for some interventions additional analyses are required to better clarify their health economic and health equity value.

## Supporting Information

S1 File This is the S1 File PRISMA checklist. (DOC)
